# Telemonitoring in Chronic Pain Management Using Smartphone Apps: A Randomized Controlled Trial Comparing Usual Assessment against App-Based Monitoring with and without Clinical Alarms

**DOI:** 10.3390/ijerph17186568

**Published:** 2020-09-09

**Authors:** Carlos Suso-Ribera, Diana Castilla, Irene Zaragozá, Ángela Mesas, Anna Server, Javier Medel, Azucena García-Palacios

**Affiliations:** 1 Department of Basic and Clinical Psychology and Psychobiology, Universitat Jaume I, 12071 Castellón, Spain; azucena@uji.es; 2 Department of Personality, Assessment, and Psychological Treatments, Universidad de Valencia, 46010 Valencia, Spain; diana.castilla@uv.es; 3 Ciber Fisiopatologia Obesidad y Nutricion (CB06/03 Instituto Salud Carlos III) (Ciber Physiopathology Obesity and Nutrition, CB06/03 Instituto Salud Carlos III Health Institute), 28029 Madrid, Spain; irenezaragoza@gmail.com; 4 Pain Clinic, Vall d’Hebron Hospital, 08035 Barcelona, Spain; amesas@vhebron.net (Á.M.); aserver@vhebron.net (A.S.); fjmedel@vhebron.net (J.M.)

**Keywords:** chronic pain, smartphone app, telemonitoring, ecological momentary assessment, randomized controlled trial

## Abstract

Background. The usefulness of mHealth in helping to target face-to-face interventions for chronic pain more effectively remains unclear. In the present study, we aim to test whether the Pain Monitor mobile phone application (app) is well accepted by clinicians, and can help improve existent medical treatments for patients with chronic musculoskeletal pain. Regarding this last goal, we compared three treatment conditions, namely usual treatment, usual treatment with an app without alarms and usual treatment with an app with alarms. All treatments lasted one month. The three treatments were compared for all outcomes, i.e., pain severity and interference, fatigue, depressed mood, anxiety and anger. Methods. In this randomized controlled trial, the usual monitoring method (i.e., onsite; *n* = 44) was compared with daily ecological momentary assessment using the Pain Monitor app—both with (*n* = 43) and without alarms (*n* = 45). Alarms were sent to the clinicians in the presence of pre-established undesired clinical events and could be used to make treatment adjustments throughout the one-month study. Results. With the exception of anger, clinically significant changes (CSC; 30% improvement) were greater in the app + alarm condition across outcomes (e.g., 43.6% of patients experienced a CSC in depressed mood in the app + alarm condition, which occurred in less than 29% of patients in the other groups). The clinicians were willing to use the app, especially the version with alarms. Conclusions. The use of apps may have some benefits in individual health care, especially when using alarms to tailor treatments.

## 1. Introduction

### Chronic Pain: A Major Public Health Challenge

Chronic pain is a multidimensional, distressing experience that can occur with or without tissue damage and persists over extended periods of time (at least three months) [1]. This disease is a public health problem worldwide [2]. Specifically, it is currently estimated that chronic pain affects between 20% and 30% of the adult population worldwide [3,4,5,6,7,8] and up to 70% of adults older than 65 years [9]. As a consequence, chronic pain has become the most expensive disease in the world [10] and accounts for up to 2% of the annual European gross domestic product [11]. Additionally, with the age distribution shifting towards the elderly [12], the economic burden of this disease is likely to increase in the coming years.

In this scenario, various efforts have been made to improve chronic pain treatments in recent decades. However, existing reviews only provide modest support for the most popular chronic pain treatments, including medical interventions, physical therapy, psychological treatment—or a combination of these [13,14,15]. Several factors, such as patient characteristics, unexplored genetic or biomechanical mechanism factors or the experience of therapists, could help explain the modest effectiveness of existing treatments for chronic pain. However, some authors have suggested that inadequate monitoring of patient progress and response to treatment is likely to be, at least in part, responsible for the limited impact of current therapies for chronic pain [16,17].

Due to its chronic nature, management of chronic pain often requires prolonged and regular contact with the health care system [18]. In this sense, it is important to note that a move towards self-management will be needed, rather than relying on the care of health professionals [19]. However, in doing so, monitoring could still remain a challenge because limited resources and existing waiting lists in public health settings limit the quality of patient monitoring in chronic pain settings [20,21]. For example, pain treatment follow-up is still predominantly discrete during on-site appointments. This is problematic because pain-related variables, such as pain intensity, mood, and fatigue can vary across and within days [22,23], even in patients with chronic pain such as osteoarthritis [24], rheumatic diseases [25], multiple sclerosis [23] and fibromyalgia [26].

The aforementioned variability of symptoms in patients with chronic pain means that a single measure may not be representative of the entire experience. Furthermore, retrospective pain assessment leads to recall bias and reduces accuracy [27]. This could be minimized with paper diaries. However, research has shown that the use of paper diaries is problematic due to participant noncompliance (missing data and back-filling) and errors associated with manual data entry [28,29]. Additionally, neither episodic on-site assessment nor paper diaries permit timely communication and response to undesired events experienced by the patient during the course of treatment [20].

Another problem related to the current model of care in chronic pain refers to decision-making in the face of unwanted events. Specifically, the current approach to care requires patients to judge when an undesired event is problematic and what is the preferred action to take in the face of that event [30,31,32]. This approach is problematic, as some patients may tolerate serious or even urgent problems (e.g., tachycardia, severe drowsiness, or persistent vomiting, diarrhea or urine retention) for too long, while others may seek care for symptoms that are less urgent or not problematic (e.g., very mild or short-term). For patients with chronic pain, an added problem is that patients combine appointments with their general practitioner, emergency services and specialized pain clinics for the treatment of their pain and related symptoms [33]. This practice is likely to be problematic, as the alternation of different specialized and nonspecialized services could lead to unpredictable treatment plans in response to unwanted events.

Telemonitoring with episodic phone calls, which is becoming an increasingly common practice, also only partially solves the aforementioned problems. First, because undesired events (e.g., side medication effects or decreased treatment effectiveness) can occur at different treatment stages [34], which means that control calls will often occur before or long after unwanted events occur. Additionally, because such phone calls require the active participation of a healthcare professional, which makes this procedure resource-consuming and ineffective [18].

Taking all the previous into account, it has been argued that our societies will not be able to sustain the current model of care for this condition [20,21,35,36], especially due to the aging of the population and the dramatic increase in the prevalence of this disease in the elderly [9]. Indeed, this appears to be true now more than ever as a result of the COVID-19 crisis, imposed restrictions on circulation and saturation of health systems [37]. Our team has already achieved some important goals in the design, development and implementation of a new tool, namely a smartphone app called Pain Monitor, which facilitates regular assessment of patient outcomes using mobile technology and minimal healthcare professional involvement in assessment. The app, which has been developed by a multidisciplinary team including physicians, nurses, psychologists and engineers following guidelines on pain research and eHealth [38,39,40,41], was found to have valid content (i.e., comparable to well-established paper-and-pencil measures) and high patient acceptability (i.e., response rates greater than 70% for daily responses over a period of one month) [42]. While important milestones have been achieved, the utility of the app in terms of increased treatment effectiveness (e.g., further reduction in pain severity and associated symptoms) remains unclear.

In fact, although it has been argued that mobile technology (mHealth)—especially the use of smartphone apps—facilitates this paradigm shift towards telemonitoring in chronic pain care, reviews on this topic have evidenced that randomized controlled trials (RCT) evaluating the usefulness of these tools are lacking in the chronic pain literature [39,41,43,44,45]. Therefore, the current investigation constitutes an important step forward into the literature on this important public health condition. In particular, the goal of the present study is to test whether incorporating the Pain Monitor app into routine medical treatment results in better pain-related outcomes in patients with chronic musculoskeletal pain. As a secondary objective, we want to investigate the opinion of healthcare professionals on the app (the patients’ opinion was already evaluated in the validation study [42]), which is key for future implementation [46].

This RCT had three conditions, i.e., usual treatment (TAU) with the usual assessment method (episodic, combined on-site and by phone call), TAU with app-based assessment without clinical alarms and TAU with app-based assessment with clinical alarms. Eligible patients were adults with chronic musculoskeletal pain, the most common chronic pain condition, which included pain in the bones, muscles, nerves, ligaments or tendons [47]. As recommended in the guidelines, we focus not only on the effectiveness of mHealth in pain severity levels [38]. In particular, outcomes also include interference of pain on functioning, fatigue and mood (depression, anxiety and anger).

The study goal is to compare the response to one month of usual pain treatment for patients in the three monitoring conditions, namely usual episodic monitoring, monitoring with an app without clinical alarms and monitoring with an app with clinical alarms. All patients received the usual treatment for their pain, so differences in outcomes (pain severity and interference, fatigue and mood) across outcomes were expected to occur as a consequence of the assigned monitoring condition. Another goal was to investigate the opinions about the app of health professionals involved in the study (e.g., in charge of disseminating the study, helping download the app and proposing the treatment).

We expected that the use of the app with alarms that were sent to the healthcare professional in the presence of unwanted clinical events would allow a quick detection of patient suffering (see the alarms in Appendix A), including severe pain levels, side medication effects, high interference of pain on functioning and psychological distress, as well as a quick reaction to these events. As a consequence of the above, we anticipated that patients in the app + alarm condition (telemonitoring) would report a greater reduction in pain severity, pain interference, fatigue, depressed mood, anxiety and anger. Additionally, we expected that patients in this condition would also experience unwanted clinical events (e.g., poor treatment response or undesired clinical events associated with treatment onset) for a shorter time compared to treatment as usual or treatment as usual with the app, but without alarms. In relation to the professionals’ opinion on the app, we expected that the professionals would experience some burden as they help patients to download the app and respond to alarms. However, we also anticipated that they would perceive the app to be useful and would be willing to use it in the future, preferably the version with alarms.

## 2. Materials and Methods

The study protocol—including a description of the planned procedures and analyses—was published before starting the recruitment [18]. However, we summarize the main study characteristics, as well as any deviations from the original plan, in the next lines.

The study started in late 2017 and ended in late 2018. All procedures were approved by the ethical review board of the Vall d’Hebron Hospital in Barcelona on 25th, June 2017 and registered on clinicaltrials.gov on 25th, July 2017 (NCT03247725).

### 2.1. Design

This is a superiority RCT with three conditions: (1) treatment as usual (TAU); (2) TAU + daily assessment using the Pain Monitor app (TAU + app); or (3) TAU + daily assessment using the Pain Monitor app with alarms (TAU + app + alarm). Because we want to ensure that telemonitoring and not the daily response to pain-related items in the app is responsible for patient benefits, we allocated patients using the app into two conditions: telemonitoring (i.e., use of the app with alarms to the physician in the presence of unwanted events detected by the app) and daily app use without alarms. Patients were randomly assigned to the study condition before agreeing to participate. Randomization was performed by an independent researcher with an online randomization tool. There was no allocation ratio. The patients’ treating physicians performed the enrollment and assignment to previously randomized conditions. Patients were assigned to conditions based on a random allocation sequence. If they refused to participate because of the assigned condition, the TAU was offered, but they were excluded from the study.

Patients and treating physicians were not blinded to allocation. In the case of patients, this was done for ethical and safety reasons, especially for those using the app without alarms, as they needed to know that the app would not report any adverse event to the physicians. Regarding the medical staff, blinding was not possible because the clinicians had to know that they would receive alarms from a subset of patients, and they had to check their electronic clinical records to decide whether a change in the treatment was required after receiving an alarm. Most assessments were completed by patients, who, as noted earlier, were not blind to their assigned condition for ethical and safety reasons. The only evaluation that was conducted by health professionals, i.e., the follow-up assessment in the TAU group, was as well not blinded. There are several reasons to explain why assessment was not blinded in this case either. For example, calling the patient required accessing to patient personal data, which is restricted to health professionals. Additionally, the TAU condition aimed at recreating the usual practice at the pain clinic, which implies that a health professional is in charge of patient episodic monitoring between appointments. All the physicians at the pain clinic participated in the study, so calls could not be performed by a clinical external to the study and blinded to the patients’ condition.

### 2.2. Participants

The study was advertised by physicians at the pain unit of the Vall d’Hebron Hospital, a tertiary care hospital in Barcelona. The study was presented to all consecutive patients with chronic pain that met the inclusion criteria. Eligibility included being over 18 years of age, not presenting any psychological disorders or problems with language that would make participation difficult, having a mobile phone with Android operating system, accepting the assigned condition and signing the informed consent form. There were no exclusion criterion in terms of previous or existing treatment for pain at study onset or treatment changes during the study, so that the study was as naturalistic as possible, and the sample representative of patients treated at the clinic. In relation to the limitation to Android operating systems, note that by the time the study was conducted the app was only available for Android for economic reasons, as this is the operating system used by more than 90% of phones in Spain [48].

Sample size was calculated a priori with the statistical software G*Power [49] considering previous studies on complications of pain treatments [30,50]. We expected that the rapid detection of such problems using alarms would result in moderate between-group differences in primary outcomes (see below). Taking 80% power, an alpha level of 0.05 and an expected Cohen’s d of 0.5, we calculated that 50 participants would be needed in each condition.

### 2.3. Interventions

All patients received the same intervention (TAU). This consisted of the usual medical treatment for their pain at the pain unit according to the usual practice at the pain clinic and pain guidelines [51,52]. The treatment could include medication (e.g., opioids, antidepressants or anticonvulsants), more invasive techniques (e.g., infiltration) or a combination of both. This treatment was proposed during the first appointment at the clinic (day of baseline assessment). However, if only an infiltration was proposed, because this could only be scheduled one or two weeks later, baseline assessment was postponed until the infiltration was performed. This was done to ensure that baseline assessment occurred on the day of initiation of treatment, regardless of the prescribed treatment.

The prescribed treatment (TAU) remained unchanged during the whole study duration (one month), except when an alarm was received by the clinicians (TAU + app + alarm condition only). Only in this scenario, phone calls were made by the physicians to explore whether a treatment change was required. Alarms were notified to the clinicians on the following working day after their occurrence. These were expected to be responded to on the same day or the next two days, depending on the severity of the alarm.

The duration of the study was set to one month since treatment onset because the clinicians considered that this is a critical period in which most problems with the treatment occur (e.g., poor effect on outcomes or side effects).

### 2.4. Pain Monitor App

Pain Monitor is a mobile phone app developed for Android, in which patients are asked to respond twice daily during 30 days (morning and evening, at flexible times from 10 am to 12 am and from 7 pm to 9 pm). A reminder is sent to the participant at 10 am and 7 pm and again 90 min later if a response had not been provided. The app incorporates different algorithms which allow sending clinical alarms to professionals (consult Appendix A to see a summary of the parameters that these algorithms follow). The alarms included in this study were established by the medical staff in a joined decision based in clinical experience and research data. The assessment protocol used in the app was validated in a previous study which allowed a reduction of items which can be more effectively used in ecological momentary assessments [42].

In accordance with recommendations from RCT guidelines in pain settings [38], outcomes included pain severity and side effects of the medication (primary outcomes), as well as fatigue, pain interference and mood states, namely depression, anxiety and anger (secondary outcomes). All these items used a 11-point numerical rating scale where 0 indicated the less severe symptomatology (e.g., no pain or sadness) and 10 reflected the most severe levels of symptoms (e.g., most intense pain or sadness). Pain interference on functioning was composed of 4 items, namely interference of pain on sleep, work/housework, leisure and social interactions. Similar to the brief pain inventory [53], an overall interference score was computed as a mean of these items to obtain a measure of overall interference of pain on functioning. For all items, with the exception of pain interference, patients were asked to report on their current status (i.e., current pain severity, fatigue and mood). For interference of pain on functioning, patients were asked to report the experienced interference during the night (interference of pain on sleep) or during the day (interference on work/housework, leisure and social interactions).

In past research, single items for each of these constructs were validated to be used in the Pain Monitor app [42]. To do so, a single item for each construct was correlated with well-established measures of pain severity, pain interference, fatigue and mood. Specifically, pain severity and interference items were validated against the brief pain inventory [53], while fatigue and mood items were validated against the Profile of Mood States [54], the hospital anxiety and depression scale for depression and anxiety [55] and the Beck depression inventory-II for depression [56]. The correlations were found to be significant and moderate in strength. The validated app items can be found in the current study protocol, which can be accessed for free [18]. For the present study purposes, only a subset of the full protocol was used. See items used in the RCT in Appendix B.

The list of adverse symptoms included the most frequent side effects of treatments for pain [52,57,58]. Note that the item that evaluated these physical symptoms did not explicitly refer to side medication effects. This was done to avoid inducing the idea that, if experienced, symptoms were due to the proposed treatment. Thus, the item stated “Please, indicate which of the following symptoms you experienced today (select only those that you do not normally experience).” Note that, even if patients experienced unusual symptoms after treatment onset, these cannot be only attributed to pain treatment, as they could be a consequence of many other factors (e.g., an infection, stress, hormonal changes, etc.).

Daily symptoms were only tracked in the TAU + app and TAU + app + alarm conditions, because these were assessed with the app. Tacking daily symptoms in the TAU group would have required daily calls, which was not feasible and would largely imply a deviation from usual practice at the clinic.

All conditions (TAU, TAU + app and the TAU + app + alarm) completed the aforementioned measures (i.e., single items on pain severity, side effects of the medication, fatigue, pain interference and mood) at baseline (the same day treatment was proposed) and the end of the study (30 days after). However, patients in the TAU + app and the TAU + app + alarm conditions also responded to the protocol twice daily. This was done to control for the effect of daily measuring (TAU + app) and to generate the alarms (TAU + app + alarm). The protocol was automatically prompted in the morning and in the evening to reduce the reliance on the patient’s memory.

Patients in the TAU condition completed the protocol as usual, i.e., with paper and pencil at baseline and by phone at the end of study (30 days later). This second measurement was not done face-to-face because patients are usually scheduled no earlier than 4–5 months after the previous appointment. In the TAU follow-up assessment, one of the pain clinic physicians, AM, made the phone calls to the participants. If patients were unavailable, they were called daily for up to three consecutive days. If no response was obtained, this was considered a missing value. The other two groups (TAU + app and TAU + app + alarms) were assessed with the Pain Monitor app at all times. The comparability of the paper-and-pencil and app assessments was not conducted here because the validity of the current assessment protocol across measurement modalities was already performed in past research [42].

The participating clinicians helped patients download the app during the initial appointment and provided support during this first use (completion of baseline evaluation). Patients also received the contact information from the lead researcher, CSR, in case technical problems occurred with the app.

In addition to patient measurement, we explored the professionals’ experience with the app. Consistent with the technology acceptance model [59], we evaluated perceived utility (“To what extent do you believe the app is useful for pain management?”), acceptability (“To what extent did you experience burden due to the alarms generated by the app?”) and intention to use (“To what extent would you like to use the app with alarms in the future?”). All physicians participating in the study and working at the pain clinic (*n* = 6) were invited to participate in this survey at the end of the study. All questionnaires were anonymized.

### 2.5. Data Analysis

First, a descriptive analysis of the sample (i.e., age, sex, marital status, job status, educational level, pain localization and pain duration), including the study flow chart, was conducted. This included an analysis of means and standard deviations for age and percentages for the remaining variables. Next, we investigated whether randomization resulted in comparable groups in terms of study outcomes (multivariate analysis of variance, MANOVA).

Originally, we planned to explore between and within changes in all study outcomes using a repeated measures MANOVA. However, during the review process it was noted that nonparametric statistics should be used due to the ordinal nature of the measures used (11-point numerical rating scales) [60]. Therefore, we calculated a Kruskal–Wallis H test to compare the change in outcomes under three conditions, as a well as a Friedman test to investigate baseline-to-follow-up changes for each condition. Both analyses were complementary, since the Kruskal–Wallis test provides evidence on between-group differences and the Friedman test reports on within-group evolution. In the Kruskal–Wallis test, the dependent variables were change scores (e.g., end of study pain severity—baseline pain severity) because the test cannot be computed with repeated measures. In Friedman’s test, analyses were computed separately for each condition because between-group changes cannot be investigated. Due to multiple comparisons, a more restrictive alpha level of 0.01 was set for the analyses.

Again different from that which was planned, we added an analysis of clinically meaningful improvements at the individual level due to insufficient power and loss in sample size (see the results section). This analysis is recommended in guidelines for pain trials and a reduction in 30% of severity of symptoms is argued to be a useful benchmark, representing at least moderate clinically significant changes (CSC) for the individuals [61,62]. Therefore, a CSC in an outcome for a given individual occurred if follow-up scores (e.g., one month after treatment, i.e., end of study) improved when compared to baseline scores by at least 30% of baseline score. For example, a baseline pain level of 5 and a follow-up pain level of 3 would represent a positive CSC reduction (the follow-up pain level is smaller than 5 − 5 × 0.3 = 3.5). Note that this 30% benchmark is only a recommended value based on past research with different outcomes and response scales, including 11-point numeric rating scales as the ones used in the present study [62]. However, change scores have ranged from 30% to 60% across investigations depending on the outcomes and the measures used [61], so the generalizability of our findings should be taken with caution.

As a final step, an analysis of variance (ANOVA) was computed to compare the number of side effects experienced by patients in the app + alarm and patients in the app without alarm conditions. This was followed by an analysis of frequencies of the types of alarms received and the clinicians’ opinion about the app. As planned, interim analyses were not conducted because no harm was expected from adding the app to TAU [18].

### 2.6. Trial Registration and Ethics

Trial registration code in ClinicalTrials.gov is NCT03247725. The ethics committee of the Vall d’Hebron Hospital approved the present study and its procedures (code PR(ATR)381/2015).

## 3. Results

**Figure 1:**
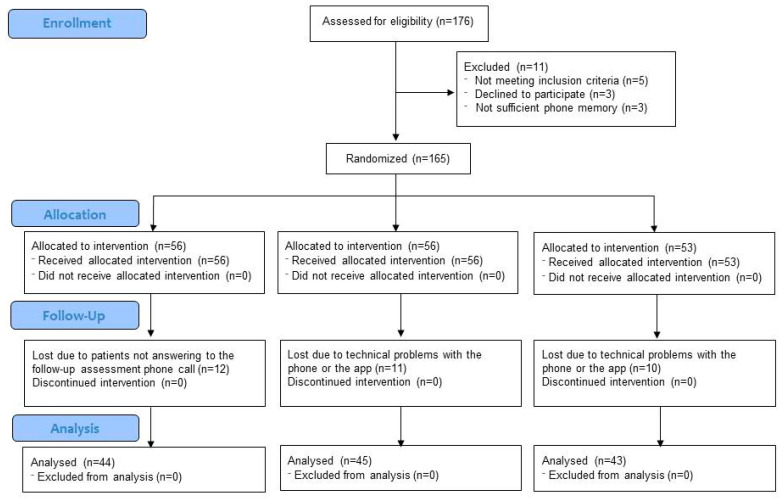
Study flowchart.

The study flowchart is shown in Figure 1. The initial recruitment plan included 150 chronic pain patients (at least 50 were needed per condition). Because we anticipated some attrition due to the longitudinal nature of the study and the occurrence of technological problems, we recruited 165 patients which were randomly assigned to conditions prior to recruitment. This resulted in 56 patients in the TAU condition, 56 in the TAU + app condition and 53 in the TAU + app + alarms condition. Finally, data were obtained from 44 patients in the TAU condition, 45 patients in the TAU + app condition and 43 patients in the TAU + app + alarm condition, which corresponds to 78.6%, 80.4% and 81.1% of the initial sample, respectively. In the TAU condition, attrition was mainly due to the fact that patients failed to respond to follow-up assessment phone calls (for each patient, up to six phone calls were made over three consecutive days to obtain the follow-up assessment). In the two app conditions, attrition was mainly a result of problems with the phone (e.g., lack of memory or buying a new smartphone during the study due to a failure or malfunction of the old one).

Overall, the response to the prompted daily questions in app was good (on average, patients responded to 77.0% of daily evaluations) and comparable to past research [42]. Note that an alarm was set to indicate when a patient was not responding to the app items (e.g., over two consecutive missing days), so that the principal investigator, CSR, could call patients and explore reasons for missing data (e.g., technical problems or lack of time or motivation).

### 3.1. Sample Characteristics

Patients had a mean age of 52.1 years (range = 23–82, SD = 11.2) and the majority were women (73.8%). Most of the participants were married or in a relationship (73.2%). One third of the patients were working at the time of assessment (34.4%). The rest of the participants were on temporary sick leave (20.6%), had compensation for permanent disability (19.1%) or were retired (11.5%), unemployed (7.6%) or homemakers (6.9%). Regarding educational level, 33.1% had only completed primary education, 32.3% had completed secondary studies, and 34.6% had completed tertiary education (technical or university studies).

Patients had musculoskeletal pain, most frequently pain in the low back or in the neck. Patients had been experiencing pain for between 6 months and 1 year (9.9%), 1–5 years (37.2%), 5–10 years (24.0%) or over 10 years (28.9%).

### 3.2. Baseline Differences in Study Variables

A MANOVA was conducted to investigate whether the randomization had effectively resulted in comparable patient profiles at baseline. The results supported the success of randomization. Specifically, baseline pain severity (F = 1.65, *p* = 0.196), pain interference (F = 1.58, *p* = 0.211), fatigue (F = 0.42, *p* = 0.656), sadness (F = 0.22, *p* = 0.806), anxiety (F = 1.01, *p* = 0.368) and anger (F = 0.42, *p* = 0.656) were comparable across conditions. The same occurred with sociodemographic characteristics, namely age (F = 2.33, *p* = 0.101), gender (χ^2^ = 1.86, *p* = 0.395), marital status (χ^2^ = 7.9, *p* = 0.660), educational level (χ^2^ = 4.72, *p* = 0.318), job status (χ^2^ = 7.59, *p* = 0.669) and pain duration (χ^2^ = 3.16, *p* = 0.789).

### 3.3. Changes in Pain Severity, Pain Interference, Fatigue and Mood across Treatment Conditions

As explained in the data analysis section and the beginning of the results section, two analytical approaches were followed due to sample size and reduced power. The first was the planned analysis of differences at the group level (Kruskal–Wallis and Friedman test). As indicated in Table 1, the Friedman test indicated that a treatment main effect was not observed for any outcome in the TAU and the TAU + app conditions. Only in the TAU + app + alarm condition a change (e.g., reduction) in pain interference was revealed (Z = −3.32, *p* < 0.001). The Kruskal–Wallis test revealed that none of the change scores differed across conditions (all *p* > 0.01).

In addition to changes at the group level, we also investigated changes at the individual level by means of an analysis of CSC (i.e., improvement of 30% or more with respect to baseline scores). The results are reported on Table 2. Again, the statistical analyses did not indicate significant differences in the proportion of clinically improved patients across conditions. However, this may again be due to insufficient sample size. Note, however, that the proportion of patients showing a CSC in outcomes was consistently higher in the TAU + app + alarm condition, with the only exception of anger, where the numbers were similar in the previous and the TAU condition. For example, 33.3% of patients in the TAU + app + alarm condition experienced a CSC reduction in pain severity after one month of treatment, while only half of this proportion improved in the other two conditions (15.4 in TAU and 16.7 in TAU + app). Similarly, a CSC in sadness was obtained by 43.6% of patients in the TAU + app + alarm condition and less than 30% of patients in the other two groups. Comparable results were revealed for pain interference, fatigue and anxiety, always in favor of the TAU + app + alarm condition.

### 3.4. Differences in Frequency of Experienced Physical Symptoms

As noted earlier, the daily frequency of undesired and unusual physical symptoms was evaluated with the app only, so these were not assessed in the TAU condition for reasons of feasibility and fidelity to usual practice. The ANOVA results indicated that the average daily number of unwanted symptoms was slightly, but not significantly higher (F = 1.49, *p* = 0.226) in the group without alarms (mean = 1.47, SD = 0.64) compared to the group with alarms (mean = 1.33, 0.38).

In total, 51 alarms were received in the TAU + app + alarm condition. The most frequent were related to recurrent headache (*n* = 11), gait instability (*n* = 10), interference of pain on sleep (*n* = 5), urine retention (*n* = 4), very severe pain levels that do not improve with treatment (*n* = 3) and sleepiness (*n* = 3). These data were collected by the app daily based on patient self-reports. In total, 93% of alarms were considered sufficiently relevant to call the patient and further investigate whether an action (e.g., a change in the medication) was required.

### 3.5. Professionals’ Experience with the App

As indicated in Table 3, the pain clinic physicians (*n* = 6) generally perceived that the app was useful for pain management (e.g., increased treatment safety and effectiveness) and for their own comfort as health care providers. Importantly, they were keener on using the app version with alarms than the version without alarms.

We also investigated burden. On average, the physicians perceived that helping the patient download the app and guiding them during the first use required 13 min (7–20 min range, median = 15 min) and responding to all daily alarms (e.g., looking at the patient’s medical record and calling them) required 28 min in total (10–45-min range; median = 27.5 min). As reported in Table 3, the burden was generally perceived as low or average.

## 4. Discussion

The present study aimed to compare the effectiveness of three monitoring approaches in patients with chronic pain, namely usual episodic monitoring, monitoring with an app without clinical alarms and monitoring with an app with clinical alarms. For one month, all patients received the usual treatment for their pain, but different effects on outcomes (pain severity and interference, fatigue, depression, anxiety and anger) were expected to emerge across monitoring conditions. Another goal was to investigate the opinion of health professionals about the app.

In relation to the first goal and contrary to our expectations, monitoring with the app and alarms did not have a significantly greater impact on outcomes compared to the usual monitoring method or the app without alarms condition, both at the group and individual level. However, consistent with our hypotheses there was only a reduction in pain interference in the group of patients using the app with clinical alarms for telemonitoring. In addition, the analyses at the individual level showed that the proportion of patients reporting a clinically meaningful reduction (i.e., over 30%) in almost all outcomes, namely pain severity, pain interference, fatigue, depression and anxiety, was higher in patients who were monitored using the app with alarms. Regarding the second objective, the healthcare professionals involved generally perceived that the app was useful for pain management in the sense that it improved treatment safety and effectiveness. They also believed that it increased their sense of comfort as healthcare providers. Importantly, they were more satisfied with the version of the app that included alarms. Regarding the burden of using the app, they reported that helping patients download the app and respond to alarms required some extra time, but they found this burden to be low or moderate.

For several years, it has been argued that eHealth and particularly mHealth will inevitably change our health systems in general [63] and pain management in particular [45]. To date, the focus of existing eHealth and mHealth solutions in pain settings has been mostly placed on self-administered treatments, mostly of psychological or alternatively of physical therapy nature [64,65,66]. While developing alternatives to face-to-face multidimensional treatments for chronic pain is indeed important, most patients still advocate individual, face-to-face treatments [67] and the first-line intervention for patients with chronic pain, namely medical treatment, will probably continue to require some patient-professional face-to-face interaction. Thus, the development of tools that improve face-to-face interventions and allow for a rapid adaptation of treatments as a function of patient evolution during treatment, as in measurement-based care [68], should be a major focus of research in chronic pain settings. This study may provide some novel insights about the potential utility, as well as the implementation challenges of this new approach to monitoring. In fact, to the best of our knowledge, this is the first randomized controlled trial that explored the utility of telemonitoring adults with chronic pain between onsite appointments using a smartphone app.

One of the findings regarding the implementation of mHealth in chronic pain management was that this system was well accepted by the pain physicians involved, which is critical for implementation purposes [69]. Importantly, they preferred the app version that included alarms, even when these generated some burden. While these results are encouraging, a lesson learned based on this perceived burden is that, if mHealth telemonitoring is to be implemented in the future, it may be advisable to allocate some time for clinicians to assist patients with the first app use, as well as to check daily alarms and call patients to make treatment adjustments. This will be especially relevant if a similar technology is to be implemented in routine care for all patients (note that, on average, it took around 30 min for each alarm, considering the time to check the electronic medical record, call the patient and make adjustments to the treatment if necessary). This time, however, clinicians found it beneficial—as indicated by reports showing overall low burden and high perceived usefulness of alarms. Additionally, these costs in terms of time spent dealing with alarms must be weighed against the potential costs of not receiving such alarms (e.g., patients attending the emergency services or taking ineffective medications). While acknowledging the previous positive findings in terms of clinician’s acceptability and the potential benefits of using alarms, it is important to note that conclusions about the actual sustainability and cost-effectiveness of this new approach to monitoring at a large scale should be taken with caution.

In relation to the utility of this new approach to monitoring, one of the main study goals was to explore whether the inclusion of telemonitoring (app with alarms) would lead to improved outcomes thanks to a rapid detection of poor treatment response, chronic low mood or high interference of pain on functioning. One problem was sample loss. Thus, even though a visual inspection of mean-level differences across conditions suggested somewhat larger improvements in the app + alarm condition in pain interference, sadness and anxiety, these results should not be overemphasized. Even the fact that pain interference only significantly improved in the app + alarm condition should be interpreted with caution due to sample size loss during the study. An interesting and more promising finding, however, was the fact that the percentage of patients reporting a clinically meaningful improvement in almost all outcomes, i.e., pain severity, pain interference, fatigue, sadness and anxiety was always higher in the app + alarm condition. This is important because, different from the analysis of changes at the group mean level, CSC is explored at the individual level. As noted in past research, while “managers and trialists may be happy for treatments to work on average, patients expect their doctors to do better than that” [70]. At the individual level, our results support the idea that the use of the app with alarms may indeed result in more patients benefiting from medical treatment. For example, while 15.4% and 16.7% of patients in the TAU and TAU + app conditions showed a clinically significant reduction in pain severity, twice the number of patients in the TAU + app + alarm condition (33.3%) showed such reductions.

An important finding in the present study was that the use of the app with alarms as adjunct to medical treatments for pain may as well provide some benefits on the functioning and mental well-being of individuals, again when explored at the individual level. Chronic pain is a very disabling and mentally distressing experience. Not surprisingly, musculoskeletal diseases, which were the type of conditions included in the present study, have become the leading cause of sick leave in Europe and account for approximately half of work-related illnesses [12] and the prevalence of anxiety and mood disorders in patients with chronic pain is two-to-three times higher than in the general population [71]. While the impact of pain on the physical functioning status and mental well-being of individuals is patent, the first-line treatments for the disease, namely medical interventions, have shown to exert a modest effect on daily functioning and, especially, on the mental health status of individuals, even when pain is effectively reduced [72,73,74]. Therefore, the finding that the use of the app with alarms resulted in a larger number of patients reporting improved pain interference with functioning, fatigue and mental well-being, is encouraging. Note for example, that depression and anxiety levels were meaningfully reduced in 43.6% and 30.8% of patients in the app + alarm condition, while reductions in depression and anxiety in the other two conditions occurred in less than 29% and 22% of patients, respectively. Again, note that these results refer to the percentage of patients with clinically significant improvements. By contrast, the analyses at the group level indicated that the effect of the condition was insufficient to reveal group differences with the recruited sample, even when pain interference only significantly improved in the app + alarm condition.

In addition to exploring the effectiveness of usual medical treatment in several conditions, this study aimed to investigate whether physical symptoms rarely experienced by the patient before treatment onset were minimized in the app + alarm condition compared to the group using the app without alarms. Of course, one thing to keep in mind is that there is no guarantee that these physical symptoms are actually due to the recently proposed pain treatment. By mentioning that patients should only report symptoms that they do not usually experience, we expected to reduce possible misinterpretation of symptoms. However, it is also possible for patients to experience unusual symptoms for reasons other than the onset of a new pain treatment (e.g., an infection, hormonal changes or a stressful daily event, to name some examples). This limitation is difficult to address, so the results regarding the number of daily symptoms should be taken with caution. While acknowledging the previous, an important finding was that algorithms can be effectively created so that the determination of when an event becomes alarming (e.g., experiencing nausea or dizziness for a given number of days) no longer depends solely on the interpretation of the patient. Of course, reporting of symptoms rather than signs requires patient self-report. However, the contribution of the present study is that an algorithm can be used to effectively send an alarm to professionals, so that action can be taken. Note that these alarms could be generated because of the ecological momentary assessment with the app, which current and past research has revealed to be feasible in terms of patient response rates [42].

As a final remark, which may be important for implementation purposes, the study revealed that patient follow-up using phone calls was difficult and technical and phone-related problems are likely to occur when implementing mHealth. In relation the former, calling patients in the TAU condition for follow-up assessment was very time consuming and largely ineffective. Specifically, if often took several calls to get in contact with the participants and these were not always available to complete the survey at the time of the call. This occurred despite the fact that patients were informed that a follow-up call would be made one month after the baseline assessment. This supports the need for more flexible and patient-dependent assessment tools, such as smartphone apps. In this sense, patient daily self-monitoring eliminated the need for physician active monitoring, so only when the app algorithm detected an undesired event, this was notified to the physician and only then a phone call was made. In relation to technical problems, such as app malfunctioning or problems with the patients’ phone, such as crashing or low battery or memory, these did occur in the study and resulted in almost 20% of loss in sample size. Recent research into telemonitoring in chronic pain states that these technical problems are something we will probably have to accept [37]. One lesson we learned, however, is that the inclusion of an alarm that sends a notification when patient responses are missing for a number of days (e.g., two in the present study) may be a good strategy to minimize technical problems. This strategy was implemented in the present investigation and it helped us identify some technical problems (e.g., patients that changed their phone or patients that were not receiving the push notifications). These problems would have probably remained undetected otherwise. Similar to past research [75], a technical team will probably be needed to address such technical problems if technological solutions are to be implemented in routine care. In addition to using alarms, another option to minimize missing data are to create a plan to maintain the app after the initial development is completed, which may help reduce technical problems associated with operating system updates [76].

To the best of our knowledge, this is the first study to use an alarm-based telemonitoring system in a randomized controlled trial in chronic pain settings. Some study limitations, including loss of sample due to technical or phone-related problems or the reduced power in the analyses have been already discussed. However, in relation to sample size it is important to mention that the anticipation of a 10% drop-out was clearly insufficient. At least 25% should have been expected based on our results. We expect that this will help researchers when calculating the required sample size for similar future studies. It is also important to acknowledge additional shortcomings, such as the reliance on subjective outcomes only (e.g., the inclusion of wearable devices could have provided more objective data on physical functioning) and the focus on musculoskeletal pain only, which prevents us from generalizing the findings to specific populations or to all chronic pain populations. In relation to wearable devices, however, it is important to note that their inclusion in routine practice may be less ecological than the use of smartphones since the latter are much more frequently utilized by the population. Another shortcoming that should be acknowledged is that the required sample size was obtained based on an uncorrected alpha level of 0.05. Corrections such as Bonferroni–Holm [77] should be considered in future similar investigations conducting multiple comparisons as this may significantly impact on sample size requirements. For instance, by correcting the alpha level to 0.01 due to multiple comparisons in the present study, the sample size needed would have increased from 150 to 222. Furthermore, in relation to sample size, it is important to note that the patient’s ability to use the technology was not evaluated in the present investigation because excellent usability findings had already been reported in the original validation study [42]. Therefore, it is difficult to know to what extent technical ability impacted on study completion and, ultimately, on the final sample size. Additionally, as mentioned earlier during the text, the selection of a 30% cutoff to determine the benchmark for a clinically significant change may also have influenced the results. It is possible that more restrictive benchmarks would have resulted in different percentages of change across conditions and, therefore, to different conclusions. The inclusion of a midpoint response, as in the “Neither agree nor disagree” option in the healthcare professionals’ questionnaire, may have also been problematic. In this sense, even though the use of a midpoint response may be useful when participants are ambivalent or neutral, they may also be used when participants have no opinion or when they want to provide a response that is socially desirable [78]. In addition, the use of midpoint responses negatively impacts score reliability [79]. In the present study, the prevalence of midpoint responses was 8.3%, which appears to be an acceptable level of uncertainty according to past research [80]. However, future research should consider whether the inclusion of such midpoint response options is crucial for the study’s needs and should preferably include a very clear midpoint response or a response scale without midpoints [78]. Finally, because no analysis of costs was made, it is difficult to know to what extent the implementation of mHealth was cost-effective. This should be addressed in future hybrid designs.

## 5. Conclusions

The present study may contribute to the field of mHealth in chronic pain in a number of ways. First, the study showed that mobile apps can be effectively implemented for patient monitoring. In this sense, the Pain Monitor app allowed us to collect a large number of data associated with pain and treatment in an automated way. This information has never before been collected with this level accuracy (i.e., daily assessment as opposed to the traditional retrospective assessment). This is important given the heterogeneity both in the etiology and the treatment of chronic pain, as ecological momentary assessment could help us obtain a more accurate picture of treatment response which may help guide interventions in a more effective manner [42]. Second, this RCT may serve guide future research in a more effective matter as it provided data on the impact that telemonitoring is likely to have on physical and mental health outcomes in patients with musculoskeletal chronic pain. Third, it showed that, if effective, telemonitoring is more likely to have an impact on outcomes if an alarm system that allows for a rapid response to unwanted events occurs, which suggests that daily assessment only is not likely to be sufficient to have an impact on outcomes. Finally, the lessons learned in terms of physicians’ burden and technology problems and solutions may help researchers, clinicians and policy makers interested in the field of mHealth.

## Figures and Tables

**Table 1:** Group-level changes in study outcomes across conditions.

	TAU			TAU + App			TAU + App + Alarm			Kruskal–Wallis Test
Outcomes	Baseline Mean (SD)	Follow-Up Mean (SD)	Friedman Z	Baseline Mean (SD)	Follow-Up Mean (SD)	Friedman Z	Baseline Mean (SD)	Follow-Up Mean (SD)	Friedman Z	Chi-Squared
Pain severity	6.28 (0.34)	5.90 (0.37)	−1.25	5.56 (0.33)	5.50 (0.36)	−0.22	5.58 (0.34)	5.14 (0.37)	−1.22	1.60
Pain interference	5.81 (0.37)	5.42 (0.38)	−0.83	4.94 (0.37)	4.75 (0.38)	−0.30	5.13 (0.37)	4.28 (0.38)	−3.32 **	4.61
Fatigue	6.41 (0.37)	5.85 (0.39)	−1.94	4.94 (0.36)	4.65 (0.37)	−1.07	5.44 (0.37)	5.14 (0.39)	−2.05	1.19
Sadness	4.26 (0.43)	4.13 (0.42)	−0.25	3.94 (0.41)	3.69 (0.40)	−0.78	4.11 (0.43)	3.47 (0.42)	−1.76	1.79
Anxiety	3.89 (0.48)	5.03 (0.45)	−2.07	3.20 (0.45)	3.50 (0.42)	−0.63	3.70 (0.47)	3.35 (0.44)	−0.75	6.55
Anger	2.73 (0.43)	3.60 (0.46)	−1.29	2.57 (0.42)	3.29 (0.45)	−1.65	2.51 (0.43)	2.60 (0.46)	−0.19	0.55

TAU—treatment as usual. ** *p* < 0.001.

**Table 2:** Percentage of participants showing a clinically significant improvement in study outcomes across conditions.

Outcomes	TAU	TAU + App	TAU + App + Alarm	χ^2^	*p*
Pain severity	15.4	16.7	33.3	4.65	0.098
Pain interference	17.5	20.0	38.5	5.27	0.072
Fatigue	17.9	14.3	25.6	1.74	0.419
Sadness	26.3	28.6	43.6	3.13	0.209
Anxiety	16.2	21.4	30.8	2.36	0.308
Anger	25.0	14.3	23.7	1.70	0.427

TAU—treatment as usual.

**Table 3:** Physician opinions about the mHealth solution.

To What Extent the App …	Completely Disagree	Slightly Disagree	Neither Agree Nor Disagree	Slightly Agree	Completely Agree
Was useful for pain management	0	0	0	3	3
Increases treatment safety	0	0	0	4	2
Increases treatment effectiveness	0	0	1	5	0
Gives me comfort	0	1	1	3	1
Is useful for me as a professional	0	0	0	4	2
Can be useful for patients	0	0	0	2	4
With alarms is something I want To use in the future	0	0	0	4	2
Without alarms is something I want to use in the future	0	2	1	2	1
Alarms impact daily job burden	0	4	1	0	1
Has an impact on burden (help patient downloading the app)	2	2	1	1	0

## Appendix A

Alarms Generated by the Pain Monitor App

- Morning pain > 7 during 5 consecutive days
- Evening pain > 7 during 5 consecutive days
- Morning sadness >7 during 5 consecutive days
- Evening sadness >7 during 5 consecutive days
- Morning anxiety >7 during 5 consecutive days
- Evening anxiety >7 during 5 consecutive days
- Vomiting during 2 consecutive days
- Tachycardia during 2 consecutive days
- Blurred vision during 2 consecutive days
- Headache during 2 consecutive days
- Dry mouth during 2 consecutive days
- Constipation during 5 consecutive days
- Drowsiness during 5 consecutive days
- Nausea during 3 consecutive days
- Itching during 3 consecutive days
- Diarrhea during 2 consecutive days
- Fever during 2 consecutive days
- Facial redness during 2 consecutive days
- Urine retention during 2 consecutive days
- Unsteady walking during 3 consecutive days
- Excessive sweating during 7 consecutive days
- Dizziness during 3 consecutive days
- Do not take the medication and there is no intention to take it
- Number of rescue medication > 3
- Sleep interference > 7
- Missing data = 2 consecutive days

## Appendix B

Items in the Pain Monitor App Used in the Randomized Controlled Trial

### Appendix B.1

Sociodemographic Items (Assessed Once, the First Day of App Use):

1. Please indicate your date of birth (DD/MM/YYYY)
2. What type of user are you?
  - I am a person with chronic pain
  - I do not have chronic pain, but I want to see the app
3. Please indicate your gender:
  - Male
  - Female
4. Please indicate your type of pain. You may select more than one option:
  - Fibromyalgia
  - Low back pain
  - Cervical pain
  - Rheumatoid arthritis
  - Osteoarthritis; Headache
  - Neuropathic pain
  - Cancer pain
  - None of the above.
5. If you selected “None of the above” please indicate your type of pain. Otherwise, leave this question blank. Press OK to continue.
6. Please indicate the location where your pain is more intense:
  - Head
  - Shoulder
  - Neck
  - High back
  - Lower back
  - Arm
  - Elbow
  - Wrist
  - Hand
  - Abdomen
  - Chest
  - Buttock
  - Hip
  - Leg
  - Knee
  - Foot
  - Whole body
  - Somewhere not listed
7. Who is currently treating your pain? You may select more than one option:
  - General practitioner
  - Rheumatologist
  - Orthopedic specialist
  - Rehabilitation physician
  - Psychiatrist
  - Pain Unit
  - Neurosurgeon
  - Neurologist
  - Oncologist
  - Another professional.
8. When did your current pain start?
  - Less than one year ago
  - Between 1 and 5 years ago
  - Between 5 and 10 years ago
  - More than 10 years ago
9. What is your current treatment for pain? You may select more than one option:
  - Physiotherapy
  - Pharmacotherapy
  - Infiltrations
  - Psychological treatment
  - Natural / alternative treatments
  - My pain is not being treated
10. Did you start a new treatment for pain in the last month?
  - Yes
  - No
11. Please select the treatment/s you started in the last month. You may select more than one option:
  - Physiotherapy
  - Pharmacotherapy
  - Infiltrations
  - Psychological treatment
  - Natural / alternative treatments
  - I have not started a new treatment
12. What is your marital status?
  - Single
  - Married
  - In a relationship
  - Divorced
  - Separated
  - Widowed
13. What is your job status?
  - Active worker
  - Sick leave
  - Permanent disability
  - Unemployed
  - Homemaker
  - Retired
  - Student
14. What is the highest level of education you have completed?
  - No studies
  - Less than high school
  - High school graduate
  - Technical training
  - University degree
15. Do you currently have a diagnosis of depression by a physician or a psychologist?
  - Yes
  - No
16. Do you currently have a diagnosis of anxiety by a physician or a psychologist?
  - Yes
  - No

### Appendix B.2

Outcomes (Assessed Twice per Day)

1. Please indicate the intensity of your CURRENT PAIN:
  0 No pain ---------10 Extreme pain
2. Please indicate the intensity of your CURRENT FATIGUE:
  0 No fatigue ---------10 Extreme fatigue
3. Please indicate the intensity of your CURRENT SADNESS:
  0 No sadness -------- 10 Extremely sad
4. Please indicate the intensity of your CURRENT ANXIETY:
  0 No anxiety ------- 10 Extremely anxious
5. Please indicate the intensity of your CURRENT ANGER:
  0 No anger ------- 10 Extremely angry

### Appendix B.3

Items Used for Generating Alarms Only (Assessed Once Daily, in the Evening Except for Pain Interference on Sleep Which Was Evaluated in the Morning Assessment)

1. Did your PAIN interfere with the quality of your SLEEP LAST NIGHT?
  0 No interference ------- 10 Maximum interference
2. Did your PAIN interfere with your SOCIAL INTERACTIONS TODAY?
  0 No interference ------- 10 Maximum interference
3. Did your PAIN interfere with the quality of your LEISURE ACTIVITIES TODAY?
  0 No interference ------- 10 Maximum interference
4. Did your PAIN interfere with your USUAL WORK or HOUSEWORK TODAY?
  0 No interference ------- 10 Maximum interference
5. Did you experience any of these symptoms TODAY? You may select more than one option. Only select symptoms that were not present before pain treatment onset:
  - Nausea
  - Vomiting
  - Tachycardia
  - Constipation
  - Drowsiness / sedation
  - Blurred vision
  - Dry mouth
  - Headache
  - None of the above
6. Did you experience any of these symptoms TODAY? You may select more than one option. Only select symptoms that were not present before pain treatment onset:
  - Dizziness
  - Itching
  - Diarrhea
  - Gait instability
  - Excessive sweating
  - Fever
  - Urine retention
  - Facial redness
  - A different symptom
  - None of the above
7. Did you take your prescribed medication TODAY?
  - Yes
  - No, but I will do it later
  - No and I do not plan to take it
  - I have not been prescribed a pain medication
8. How many times did you take a rescue medication TODAY?
  - 0
  - 1
  - 2
  - 3
  - 4
  - 5
  - 6
  - 7
  - 8
  - 9
  - 10
  - More than 10
